# Supplementary material for: Applying height differentiation of tactile symbols to reduce the minimum horizontal distances between them on tactile maps
Source: PLoS One. 2022 Feb 25;17(2):e0264564. doi: 10.1371/journal.pone.0264564 (PMC8880946; doi:10.1371/journal.pone.0264564)
Supplement: S1 File — Formal description of the testing phase. (DOCX) [file pone.0264564.s001.docx]

# S1 File. Research Protocol.

## Recruitment phase

A group of 25-30 adults (age 15-65) who are blind or visually impaired will be recruited to participate in the study. Participants will be asked to examine tactile stimuli. This number of participants allows avoiding potential confounds related to the repetition of location tasks on a number of variants of the same map (controlled order). Due to the nature of the study, participants must be able to use both hands for the map scanning process and comprehend oral explanations in Polish or English. As our goal is to verify the research hypothesis in depth, we would like to invite participants of various experience level of working with tactile maps and braille literacy proficiency (participants will be asked to determine their experience in this matter during application process). However, braille literacy is not required. We would like to recruit both congenitally and adventitiously blind individuals.

The recruitment process will be carried out by the Research team. Recruitment forms will be disseminated by both the Research team members and Polish Blind Association (<https://pzn.org.pl/>). The overall nature of the study will be described in the recruitment form. Potential participants will be informed that their behaviour during the study would be video recorded. People willing to take part in our research study will indicate their interest through a web-based response form – candidates will be asked to fill a simple application to confirm their eligibility for the study. The research team members will be available to view the forms filled by candidates. Eligible candidates will be chosen to provide the most diverse group possible considering age, type of blindness, level of experience working with tactile maps and braille literacy. Participants will be compensated for their time at a rate of 100 PLN per hour. We anticipate the study sessions to last no more than 90 minutes.

## Testing phase

Participants will be given a written informed consent to participate [3 minutes].

1. If a participant agrees with the consent, one of the researchers will begin video recording process.
2. Participant will be presented with a pilot map stimulus. Each participant, prior to performing the designed tasks, will be allowed to examine the map briefly – explore its dimensions and material used as well as signs used to present particular real-world elements using the associated map legend [5 minutes].
3. One of the researchers will introduce every participant to the map topic and briefly describe tasks to be solved by the participants [3 minutes]. Below is an exemplary introduction:

*“Right in front of you there is a Captain Flint’s treasure map. Please refrain from examining the map’s content before asked to do so. This map shows the path that needs to be taken from your current location to the treasure buried long time ago by the famous pirate. The treasure, however, is well protected and the path itself is dangerous. There are areas of moving sands, deep water reservoirs and pirate camps that you should avoid. Many roads and paths are crossing the map but only one of them is the safe way to reach the treasure. The captain has also placed on the map features that facilitate orientation, such as trees. All these things are presented on the map in a form of points, lines and patterns that are combined in various ways. Your tasks will be related with some of the objects just mentioned. I will ask you to find specific features on the map.”*

*“You will be asked to repeat these tasks on six different variants of the map. They have different arrangement of features on them and their design slightly differs.”*

1. After “introduction” there will be some time for questions from participants in case of any ambiguity [5 minutes].
2. Next step will be the actual testing phase. Participant will be introduced to each of the three spatial tasks to be solved [2 minutes each]. Consecutive descriptions will be presented after solving the previous task. The maximum time anticipated for solving each task is 5 minutes [maximum 15 minutes total for each map variant].

Below are exemplary task descriptions and testing procedures:

***TASK 1 (point signs):*** *“Captain Flint was a very cautious pirate. He wanted to protect his treasure by all means. This is why he set 5 traps in its surroundings that might activate when stepped on. These traps are represented by the circle symbol (placed in the legend). You can get back to this reference at any time but please take your time now to examine it thoroughly and learn its shape and how it feels while touched.”*

*“When you are ready, please locate 5 trap locations presented on the map. I will measure the time necessary for you to solve the task. Let me know anytime you find a trap sign on the map by tapping your finger and confirming this fact vocally.”*

*(start the timer and stop it after the task is solved or when the time limit is reached)*

***TASK 2 (areal signs):*** *“Not only was the captain very cautious but also a very smart pirate. When choosing location to bury his treasure he considered also natural obstacles. The treasure is surrounded by 5 areas of moving sands that are depicted on the map using line pattern (placed in the legend). Take your time to study the pattern presented. You can get back to this reference at any time but please take your time now to examine it thoroughly to learn its shape and how it feels while touched.”*

*“When you are ready, please locate 5 areas of moving sands on the treasure map. I will measure the time necessary for you to solve the task. Let me know anytime you find one of the areas of moving sands by tapping your finger and confirming this fact vocally.”*

*(start the timer and stop it after the task is solved or when the time limit is reached)*

***TASK 3 (line sign):*** *“Fortunately, regardless of all precautions taken by Captain Flint, with this map in possession, you are able to safely reach the treasure. Your location and the treasure are connected by a path. These features are represented by the square symbol (placed in the legend). You are at the beginning of the path; the treasure is on its other end. Bear in mind that the path curves in many different directions and might be interrupted by symbols representing other features. In a while I will show you where the point indicating your starting location is.”*

*(tell a participant, where the starting point is)*

*“When you are ready, please follow the path leading from it until you reach the treasure. I will measure the time necessary for you to solve the task. Let me know when you find the treasure’s location.”*

*(start the timer and stop it after the task is solved or when the time limit is reached)*

1. After completing all three tasks on one of the map variants, a participant will be asked to repeat the same tasks on consecutive variants (six variants, up to eighteen location tasks total). The order in which particular map variants will be presented is going to be controlled in a way to avoid confounding variables. For example, participant A will get map variant 1 as the first stimulus to investigate, while participant B will get map variant 5 as the first stimulus to work with. The task description will be repeated upon request of the participant and is not meant to be repeated before every map variant.

The times required for solving particular location tasks will be noted (stopwatch results - digital spreadsheet: expressed in seconds). Any cases of tasks unsolved within the time limit will be noted (digital spreadsheet) along with errors committed (e.g. confusing 2 point symbols). The times recorded for solving particular location tasks will be analysed using significance tests. Demographic details of the participant population will be reported in aggregate. All the results and any quotations will be presented anonymously in the reports.

A number of questions is going to be asked at the end of a session to evaluate participants’ feelings regarding the presented stimuli – qualitative study. The answers will be saved but not associated with specific participants (the participant’s familiarity with tactile maps and type of blindness, however, might be noted). Exemplary questions are listed below:

- At what age did you lose vision (age)?
- How often have you used tactile maps so far?
- Was it easy to understand the content of the maps?
- Was using the maps comfortable?
- Did you notice how the consecutive map variants differed? What was it?
- Do you have any suggestions on how to improve these and similar tactile maps?

## Informed consent process and participant privacy

Upon arrival at the session, each participant will be individually and in-person introduced to the study and provided with a copy of the informed consent document in paper form (digital, screen reader accessible form will be provided upon request). One of the researchers will offer to either read the consent form aloud or let a potential participant read it himself/herself. Potential participants will be noticed that the session is going to be video recorded (this information will also be mentioned in the recruitment announcement), but their identity will not be known to other participants nor anyone else apart from the researchers themselves. The consent form will also ask the participants if they agree to take and publish photos of them during the tests in scientific articles and/or research reports (this consent is not mandatory). As part of the informed consent process, participants will be asked to consent to the public release of data collected during study session after their de-identification using standard procedures. These might include individual results across tasks performance and committed errors.

After reading, one of the researchers will offer to answer any questions. Participant will be then asked if he/she is willing to take part in the study. If so, they will be asked to sign a copy of the informed consent document (signature guides will be provided).

Potential participant’s personal data, such as: name, age, experience with tactile maps, screen readers and braille literacy proficiency (four-point scale: none, low, average, high), type of blindness (congenital, adventitious), contact information (e-mail address and phone number), preferences regarding session day and time will be gathered. The preferred contact method will be used to contact the respondents to provide additional information (as requested by the respondent), answer questions (asked by the respondent), or schedule testing sessions.

Video files will be recorded during sessions with each participant. These videos will not be published but only used to draw conclusions regarding participants’ behaviour during the tasks (e.g. stimuli scanning strategy) for future research. The video material will also be used to confirm and/or verify all the measurements taken during the study (e.g. times to solve particular tasks).

All the results and notes taken during sessions will be stored in a form of MS Excel spreadsheets. Exemplary answers to the post-study questionnaires might be quoted in open-access materials but they will not provide any participant’s personal data.

The sessions will be conducted in a reserved and closed room but the surrounding might be open to public access. One participant will be in the room at a time. One or two researchers will conduct the study. After every study session the room and the stimuli will be disinfected to prevent the risk of COVID-19 spread.

Each participant will be assigned a numeric code that will be used to label the data collected during their sessions. We will also assign participants’ responses regarding their experience with tactile maps and Braille, as well as type of blindness (congenital, adventitious) to the specific numerical codes. Informed consent in a form of single document that will associate the participants’ names with their numeric codes will be stored in a faculty office (locked cabinet).

All the data obtained during sessions will be password protected and shared only between selected research team members. The data might be backed up to password-protected external data storage (such as flash memory drive). The records will be kept for publication purposes and potential re-use in the future in an unaltered form.

After taking part in the study, participants will sign the appropriate documents to receive their renumeration. The receipts will be prepared by the Polish Blind Association, who will be responsible for maintaining the documentation.
