# Supplementary material for: Applying height differentiation of tactile symbols to reduce the minimum horizontal distances between them on tactile maps
Source: PLoS One. 2022 Feb 25;17(2):e0264564. doi: 10.1371/journal.pone.0264564 (PMC8880946; doi:10.1371/journal.pone.0264564)
Supplement: Attachment — Submitted filename: Response to Reviewers.docx [file pone.0264564.s002.docx]

Dear Editor, Dear Reviewers

We are very grateful to have been given the opportunity to revise our manuscript entitled “Applying height differentiation of tactile symbols to reduce the minimum horizontal distances between them on tactile maps” for the PLOS ONE journal (Manuscript ID: PONE-D-21-29348).

We would like to thank the two anonymous Reviewers for their highly valuable comments, which provided useful suggestions that helped us extend the content of the article and correct some of its issues. We have carefully analyzed the comments and suggestions provided by the reviewers. Detailed responses to each point raised by the academic editor and reviewers are available in the following pages.

The manuscript has been revised according to your recommendations in a ‘track changes’ mode. It includes a number of changes, among which the most important are:

- We have included more detailed descriptions of stimuli variants and the dedicated figure.
- A priori hypothesis regarding the RQs 1 and 2 has been described.
- Figures and tables numbering has been updated.
- Additional statistical analysis verifying the impact of type of blindness and level of experience with tactile maps/braille reading has described inserted in the manuscript.
- The discussion section has been extended to clarify the impact of our work on tactile maps standardization.
- The word *sign* has been replaced with *symbol*.
- The abbreviation PVI (people with visual impairments) has replaced the previously used one: BVI (blind or visually impaired).
- The order of elements in Table 1 has been updated.
- Minor editorial changes, e.g. typos and spelling.

Once again we would like to thank you for your insightful comments. We did our best to be responsive to them. We hope that the revisions have improved the paper and made it suitable for the publication in the PLOS ONE journal. Thank you for your time and effort.

Jakub Wabiński

Military University of Technology

Warsaw, Poland

**The Academic Editor**

| **No.** | **Reviewer Comment** | **Authors’ Response** |
| --- | --- | --- |
| 1 | Please ensure that your manuscript meets PLOS ONE's style requirements, including those for file naming. The PLOS ONE style templates can be found at  https://journals.plos.org/plosone/s/file?id=wjVg/PLOSOne_formatting_sample_main_body.pdf and  https://journals.plos.org/plosone/s/file?id=ba62/PLOSOne_formatting_sample_title_authors_affiliations.pdf | We have carefully read the guidelines and used the style templates provided. We hope that the manuscript meets PLOS ONE’s style requirements. |
| 2 | We note that Figure 6 includes an image of a participant.  As per the PLOS ONE policy (http://journals.plos.org/plosone/s/submission-guidelines#loc-human-subjects-research) on papers that include identifying, or potentially identifying, information, the individual(s) or parent(s)/guardian(s) must be informed of the terms of the PLOS open-access (CC-BY) license and provide specific permission for publication of these details under the terms of this license. Please download the Consent Form for Publication in a PLOS Journal (http://journals.plos.org/plosone/s/file?id=8ce6/plos-consent-form-english.pdf). The signed consent form should not be submitted with the manuscript, but should be securely filed in the individual's case notes. Please amend the methods section and ethics statement of the manuscript to explicitly state that the patient/participant has provided consent for publication: “The individual in this manuscript has given written informed consent (as outlined in PLOS consent form) to publish these case details”.  If you are unable to obtain consent from the subject of the photograph, you will need to remove the figure and any other textual identifying information or case descriptions for this individual | As we were unable to obtain a written consent on the journal’s form (we collected a written consent form on our own template during the study), we have modified the figure so that the participant could not be identified. The figures numbering has been modified and thus, the figure is now saved under Fig 7. |

**Reviewer #1**

| **No.** | **Reviewer Comment** | **Authors’ Response** |
| --- | --- | --- |
| 1 | In this manuscript, the understanding of the tactile map was improved by the height differential signs. In addition, it was suggested that the height differences can reduce the recommended minimum horizontal distance between signs on the tactile map, and provide more relevant information or simply make the map easier to read. I think that this manuscript is acceptable for publication after minor revision. | Thank you for your appreciation. |
| 2 | Three tasks are performed in this study. but, the task numbers are not shown in "Human subject testing". I suggest to you that you write as follows in lines 237 to 242: "Task 1. Locate 5 point symbols ...", "Task 2. Locate 5 area symbols ..." and "Task 3. Follow the path ...". | Thank you for this suggestion. The text has been modified according to the reviewer’s comment. |
| 3 | The table numbers quoted in the document are incorrect as follows:  Line 351: “table 4-6" --> "table 3-5".  Line 392: "Table 7" --> "Table 6".  Line 402: “tables 8-10” --> “table 7-9”.  Line 501: “Table 3” --> “Table 10”. | Thank you for this suggestion. Tables numbering has been updated. |

**Reviewer #2**

| **No.** | **Reviewer Comment** | **Authors’ Response** |
| --- | --- | --- |
| 1 | The transformation of digital map into tactile form is an important issue in efforts to compensate information for the visually impaired, and creating a tactile map will require a certain amount of standardization. This paper tested weather cartographic signs on tactile maps to different heights can reduce the minimum horizontal distances between them. This test will lead to efficient production of tactile maps. However, there are a few concerns about the testing. | Thank you for your appreciation. We have carefully read your comments and did our best to clarify all your concerns. |
| 2 | The authors used 6 different stimuli that have different horizontal distances between tactile signs (D1, D1HD, D2, D2HD, D3, D3HD). It is better to show what all the stimuli look like in the Figure, and to propose hypothesis in advance about what we can learn by comparing what with what regarding three Research Questions proposed by the authors. | Thank you for this suggestion. The appropriate figure has been included in the manuscript (Fig 2).  The a priori hypothesis has been added in the manuscript. Additional description is now also present (lines 161-166). |
| 3 | - It seems predictable that the performance would be better with height difference, and… - …the stimuli used in this experiment seem arbitrarily chosen. - It is not clear how they will contribute to standardization of tactile map directly. | - Indeed, this sound apparent but it turns out that applying height differentiation of symbols on tactile maps is uncommon (according to the questionnaire results that we have recently carried out for one of our parallel studies), mainly due to the limitations of the production techniques used. - We have done everything possible to avoid potential confounding variables in our study. There is a paragraph describing this process (Lines 167-171). We have also emphasized the fact that the stimuli variants were presented to the study participants in random order. - Thank you for this comment. We have added 2 paragraphs the in Discussion section to clarify our statement (lines 565-581). |
| 4 | As for Performance results, from my understanding, the comparisons between D1-D1HD, D2-D2HD, and D3-D3HD are essential. Why are the authors making statistical comparisons even for unrelated combinations? The statistical descriptions seem to have become cumbersome. | The aforementioned paragraph (lines 161-166) explains why we needed to analyze all of the variants and include them in the statistical analysis – providing answers to the RQs 1 and 2. We did our best to simplify the presentation of statistical analysis to ensure smooth reading. |
| 5 | Is there any difference in performance between people who are congenitally and adventitious blind? If so, the analysis is performed separately. | That is a very good question. We have performed additional statistical analysis of this issue. It turns out that there are no statistically significant differences between congenitally/early and adventitiously blind. The same is true for participants with different levels of expertise in braille reading and tactile maps use. We have included a paragraph describing this analysis (lines 373-377). |
